# Hyperdimensional Analysis of Amino Acid Pair Distributions in Proteins

**DOI:** 10.1371/journal.pone.0025638

**Published:** 2011-12-09

**Authors:** Svend B. Henriksen, Rasmus J. Mortensen, Henrik M. Geertz-Hansen, Maria Teresa Neves-Petersen, Omar Arnason, Jón Söring, Steffen B. Petersen

**Affiliations:** 1 NanoBiotechnology Group, Department of Physics and Nanotechnology, Aalborg University, Aalborg, Denmark; 2 International Iberian Nanotechnol Lab (INL), Braga, Portugal; 3 Nanobiotechnology Group, Department of Biotechnology, Chemistry and Environmental Sciences, University of Aalborg, Aalborg, Denmark; 4 Nanobiotechnology Group, Department of Health Science and Technology, Aalborg University, Aalborg, Denmark; 5 The Institute for Lasers, Photonics and Biophotonics, University at Buffalo, The State University of New York, Buffalo, New York, United States of America; University College Dublin, Ireland

## Abstract

Our manuscript presents a novel approach to protein structure analyses. We have organized an 8-dimensional data cube with protein 3D-structural information from 8706 high-resolution non-redundant protein-chains with the aim of identifying packing rules at the amino acid pair level. The cube contains information about amino acid type, solvent accessibility, spatial and sequence distance, secondary structure and sequence length. We are able to pose structural queries to the data cube using program ProPack. The response is a 1, 2 or 3D graph. Whereas the response is of a statistical nature, the user can obtain an instant list of all PDB-structures where such pair is found. The user may select a particular structure, which is displayed highlighting the pair in question. The user may pose millions of different queries and for each one he will receive the answer in a few seconds. In order to demonstrate the capabilities of the data cube as well as the programs, we have selected well known structural features, disulphide bridges and salt bridges, where we illustrate how the queries are posed, and how answers are given. Motifs involving cysteines such as disulphide bridges, zinc-fingers and iron-sulfur clusters are clearly identified and differentiated. ProPack also reveals that whereas pairs of Lys residues virtually never appear in close spatial proximity, pairs of Arg are abundant and appear at close spatial distance, contrasting the belief that electrostatic repulsion would prevent this juxtaposition and that Arg-Lys is perceived as a conservative mutation. The presented programs can find and visualize novel packing preferences in proteins structures allowing the user to unravel correlations between pairs of amino acids. The new tools allow the user to view statistical information and visualize instantly the structures that underpin the statistical information, which is far from trivial with most other SW tools for protein structure analysis.

## Introduction

Proteins attain their function through their folded 3D structure and to date 1288 different folds have been identified [1], [2]. The protein fold is a cumulative result of numerous interactions between amino acid residues interacting with each other through space and/or chemical bonds. These include disulphide bridges and non-bonding interactions, such as salt bridges, hydrogen bonds and hydrophobic interactions [3], [4]. The three dimensional fold of a protein sequence is achieved through optimization of a hierarchical set of rules, reflecting closest possible packing of the polypeptide chain and simultaneously positioning of hydrophobic and charged residues [5]. Several parameters influence the contribution of the amino acid pair interaction to the folded protein stability. The solvent accessibility of each amino acid plays a major role in the pair's interaction energy, and therefore on the protein stability. The secondary structural element where each amino acid is located as well as the pair's spatial and sequence distance will also influence the contribution of such pair to protein stability. We interpret the interaction between two amino acid residues in terms of 8 parameters: the type of each amino acid residue interacting (AA1, AA2), their solvent accessibility, the secondary structural element where they are located (SS1, SS2), the protein size, the sequence and spatial distances between the amino acid residues interacting. The 8 dimensional data cube represents our perception of protein fold space.

Several relevant works addressed the rules of packing amino acid residues in proteins [6]–[14]. Applying methodologies for finding correlated pairs of residues has always been of interest to protein science. Such correlations usually arise from direct close spatial interactions between residues, although allosteric effects may result in correlations between distant residues. The utility of multiple sequence alignments based approaches for detecting correlated amino acids has been known for almost two decades [15]–[17], and improved methods are being developed [18]. However, their usage by the scientific community has been limited due to lack of access to theoretical and computational approaches as open source tools or through user friendly interfaces. Only a few programs have been implemented to date on the web [19]–[21]. Some programs can be downloaded for local usage, such as PlotCor [22], CorrMut [19] and CRASP [23]. CysView is a web-based application tool that displays cysteine connectivity patterns in proteins [24]. ESBRI is a web tool which analyses the salt bridges in a protein structure [25]. However, the general absence of graphical analysis tools makes it difficult to analyze amino acid pair interactions and examine them with respect to structural data. Most amino acid pair interaction data presented in literature appears as 1D or 2D plots, thus effectively being projections of the total fold space onto a 1D or 2D subspace. No currently available method allows the user to view statistical information and visualize instantly the pdb structures that underpin the statistical information.

**Figure 1:**
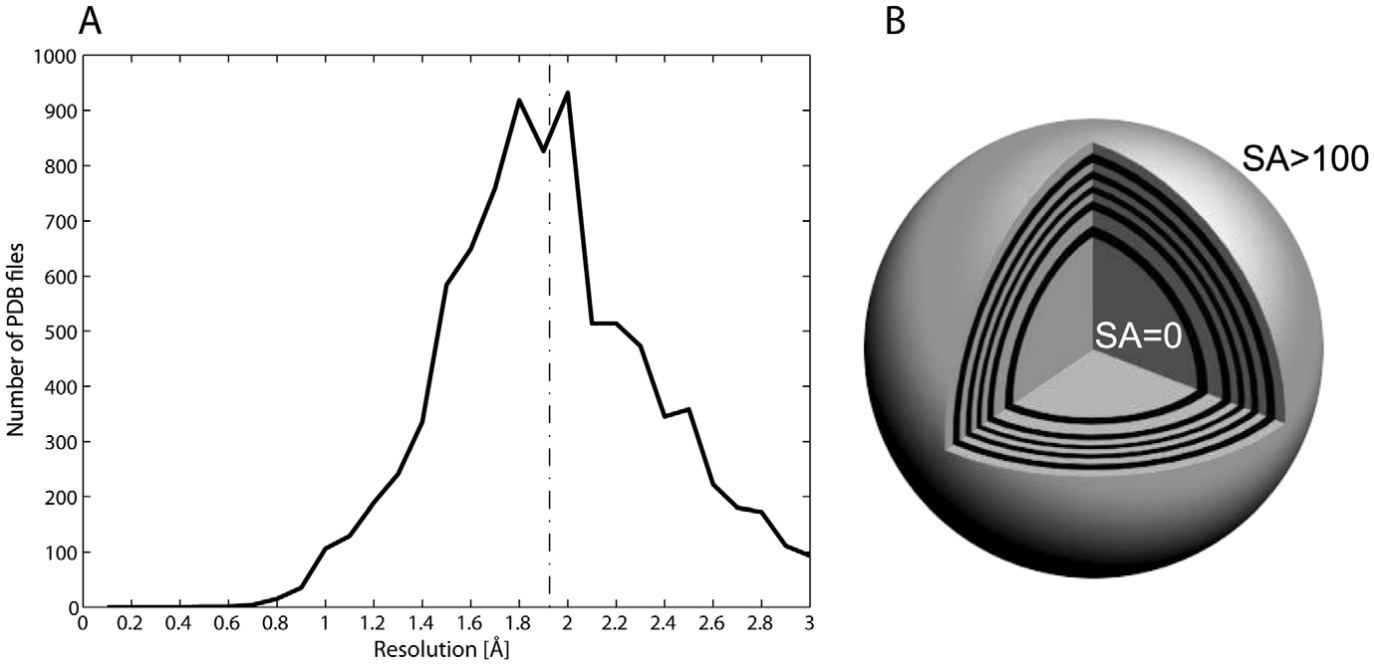
Protein chains resolution and solvent accessible shell concept. A) Histogram of the resolution of the 8706 protein chains used in this study. The average resolution is 1.93 Å as indicated by the dotted line. B) Spherical model of a globular protein displaying the solvent accessible shell concept: the protein residues are binned in solvent shells ranging from completely buried (SA = 0%) to full solvent exposure (SA = 100%) in steps of 10%, with 0% and >100% treated explicitly.

In the present study we define each pair of amino acids in terms of (number of bins used in brackets): amino acid type ×2(20), solvent accessibility (12), spatial distance (14), secondary structure ×2(4), protein size (12) and sequence distance (6). We only consider a pair if the two amino acids are located in the same solvent accessibility bin and if the inter residue distance is less than 8.3 Å. The resulting 8 dimensional fold tensor contains ∼77.4 million cells. In our analysis of 8706 protein high resolution 3D chains (Fig. 1A), ∼5.9 million amino acid pair observations were found and loaded into ∼1.9 million cells in the fold tensor. Each of these cells contains the number of times a pair of two particular amino acids has been found at a location in fold space. The volume of a protein has been divided into eleven spherical layers of solvent accessibility. This concept is illustrated in Figure 1B. The presented programs ProExtract, ProPack and ProPair successfully identify known structural motifs and show their potential for finding novel packing preferences in proteins. In order to demonstrate the capabilities of this general data cube as well as the query programs we have studied the packing of disulphide bridges as well as charged amino acid pairs. These choices represent only a very small fraction of the types and number of queries that ProPack and ProPair would allow. Previous studies report the geometrical configuration and/or statistical characterization of amino acid residues participating in disulphide bridges [26]–[43] and salt bridges [44]–[48]. The present paper presents a bioinformatics approach that allows the user to carry out hyper dimensional analyses of amino acid pair interactions and their distribution in proteins with an incorporated graphical analysis tool, making possible the visualization of any conceivable combination of the 8 dimensions for each amino acid pair. Any interacting amino acid pair of interest can be visualized in its structural location within a particular structure. We aim at making these programs available on the web.

## Materials and Methods

According to a recent comprehensive review [49], to establish a really useful statistical predictor (or model) for a protein system, we need to consider the following procedures: (i) construct or select a valid benchmark dataset to train and test the predictor; (ii) formulate the statistical samples with an effective mathematical expression that can truly reflect their intrinsic correlation with the attribute to be predicted or analyzed; (iii) introduce or develop a powerful algorithm (or engine) to operate the prediction or analysis; (iv) properly perform cross-validation tests to objectively evaluate the anticipated accuracy of the predictor; (v) establish a user-friendly web-server for the predictor that is accessible to the public. In this section we will describe how to deal with these steps.

In statistical prediction, the following three cross-validation methods are often used to examine an analysis method or predictor for its effectiveness in practical application: independent dataset test, sub-sampling (5-fold or 10-fold cross-validation) test, and jackknife test [50]. As elucidated in Ref [49], among the three cross-validation methods, the jackknife test is deemed the least arbitrary that can always yield a unique result for a given benchmark dataset, and hence has been increasingly used and widely recognized by investigators to examine the accuracy of various predictors [51]–[55]. In our case, we are not attempting prediction. Instead we are extracting statistically significant data from a large set of experimental observations.

### Protein Dataset

In order to avoid homology bias and remove the redundant sequences from the benchmark dataset, a cutoff threshold of 25% should be used [49], [56]. However, in this study we did not use such a stringent criterion because the currently available data do not allow us to do so. Otherwise, the numbers of proteins for some cases would be too few to have statistical significance. A list of high resolution protein chains (resolution ≤3.0 Å) with sequence identity ≤35% was retrieved from the Pisces server [57]. All structures had a minimum chain length of 40 and a maximum R value – a measure of how well the experimental data can be predicted from the refined model - of 1.00. Non-X-ray structures and structures only with Cα atoms were excluded. The Pisces culling method selected was “chain”. The downloaded list contained 9039 chains, present in 8598 different .ent files. The .ent files were downloaded from the Research Collaboratory for Structural Bioinformatics (RCSB) [58]. The corresponding .hssp files were downloaded from the homology-derived secondary structure of proteins (HSSP) database [59]. Entries in the Pisces list for which the corresponding .hssp files were not available were discarded, leaving 8272 .ent files with corresponding .hssp files. These files contained 8706 of the non-redundant chains from the Pisces list.

### Software

**Figure 2:**
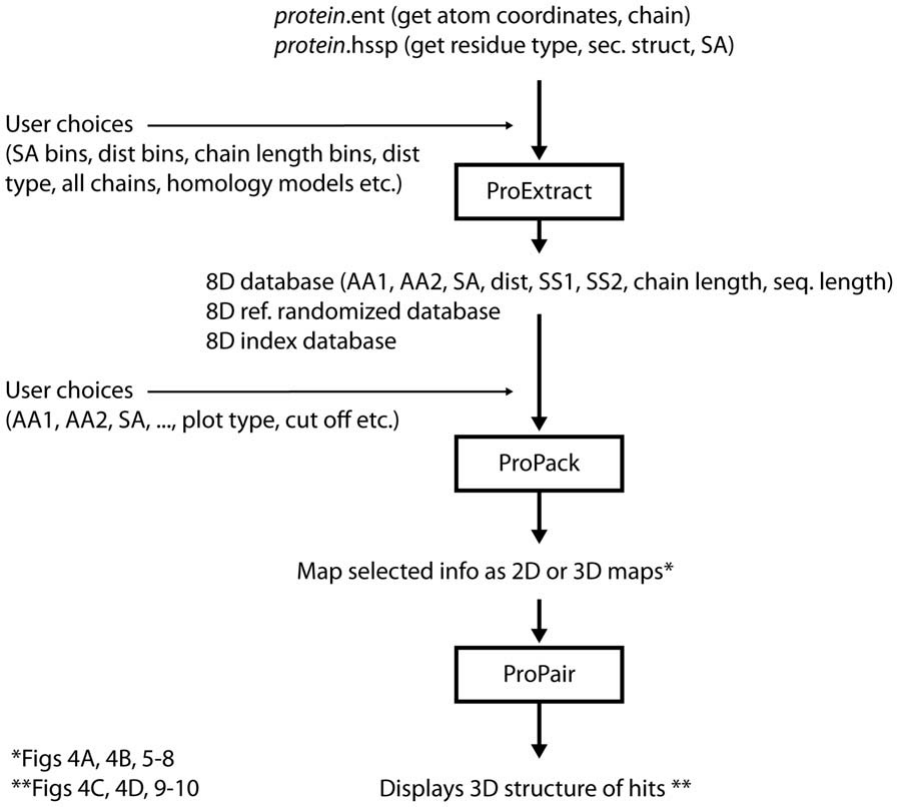
Flowchart depicting the main programs built in order to carry out the presented data as well as the input and output files needed by each program. AA1 is amino type 1, AA2 amino type 2, SA is the solvent accessibility of the protein shell where the selected amino acid pair is located, Dist is the spatial distance between the selected amino acids, SS1 is the secondary structure of AA1, SS2 is the secondary structure of AA2, sequence distance is the primary sequence distance between the amino acid residues selected, chain length is the length of the chain being analyzed.

Three software packages were developed: ProExtract, ProPack and ProPair (Fig. 2). In addition specific programs were written to define the solvent shells as well as amino acid solvent accessibility distributions. All programs were developed using MATLAB v7 (2010a) [60]. The source code of the programs ProExtract (used to create the 8D tensor), ProPack (the query program that allows the user to access the 8 dimensional data tensor) and ProPair (the query program that allows the user to select a particular set of cells in the data tensor and which retrieves a list of proteins with specific pairs) have been uploaded as supplementary information. The file names are:

#### ProExtract files

“ProExtract_V2p4.m”, “ProExtract_V2p4.fig” to be found in Figure S1.

#### ProPack files

“ProPack.m”, “ProPack.fig” to be found in Figure S1.

#### ProPair files

“PairFinder_v1p2.m”, “PairFinder_v1p2.fig”, “PairSearcher_v1p1.m” to be found in Figure S1.

The description on how to run the software ProExtract, ProPack and Propair can be found in Figure S1: “ProExtract_User instructions.doc”, “Propack_User instructions.doc, “ProPair_user instructions.doc”, respectively.

As mentioned in Figure 2, in order to run ProExtract, two input files are needed: the protein.ent list and the list of correspondent hssp files. A file named “pisces_35_id_files_with_hssp.txt” has been uploaded as supplementary information (please open uploaded file Figure S1) where the name of all pdb files has been listed. This file should be open with WordPad. The associated .ent and .hssp files are publically available.

### Solvent shell

Each protein was treated as being made of shells with different solvent accessibilities. The coordinates of all atoms were extracted from the corresponding .ent file and used to calculate the geometric midpoint (M) of each residue:
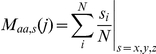
where *N* is the number of atoms in residue number *j*, and *s* is the *x*, *y* and *z* coordinates of atom number *i* in residue *j*. The geometric midpoint of all residues was calculated by:
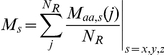
where *N_R_* is the number of amino acids in the protein. This defined the geometric midpoint of the protein, which is the center of the protein when assuming that the protein is globular. The Euclidian distance from this center to the geometric midpoint of each amino acid residue was then calculated.

The solvent accessible surface area of each residue was read from the corresponding .hssp files and converted to a percentage of solvent accessible area by dividing the value with the total surface area of the residue side-chain (calculated from a Gly-X-Gly tripeptide [61]). The secondary structure information of each amino acid was also extracted. The protein residues were binned in solvent shells ranging from totally buried to full solvent exposure in steps of 10%, with 0% and >100% treated explicitly. The average distance to the protein center was calculated for all residues in every bin, thereby obtaining a “thickness” of each solvent accessible shell (Fig. 1B). This can only be considered as an approximation since many proteins differ significantly from spherical structure.

### Amino acid solvent accessibility distributions

**Figure 3:**
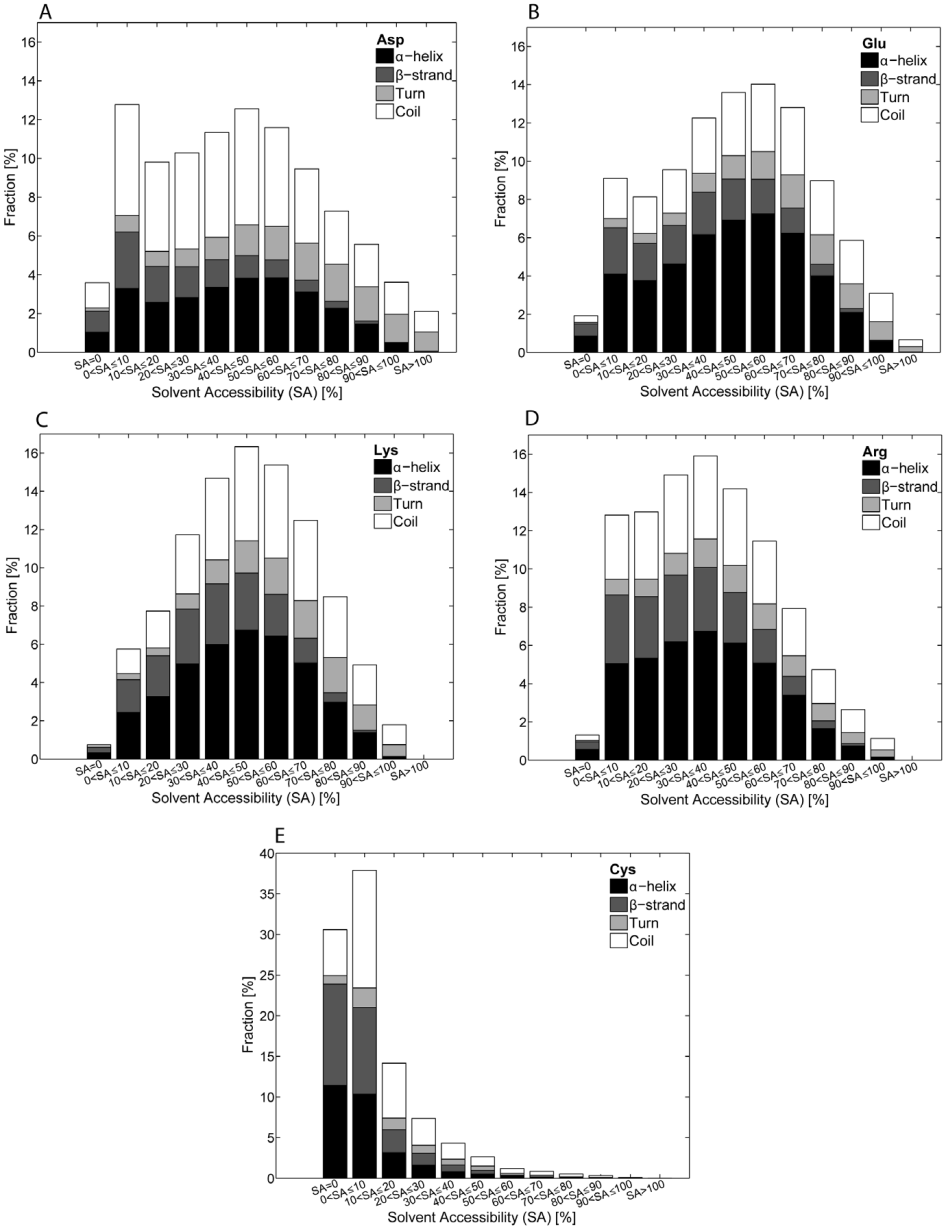
Histograms displaying the abundance of selected amino acid residues (Asp, Glu, Lys, Arg, Cys) in each protein shell characterized by a specific solvent accessibility SA. For each SA bin is also displayed the relative abundance of each selected residue in a particular secondary structural element (α-helix, β-strand, turn and coil). The first and last bins contain the fraction of residues in completely buried protein shell (0% SA) and in a completely solvent accessible protein shell (100% SA), respectively. A) Distribution of 120553 Asp residues, B) Distribution of 141961 Glu residues, C) Distribution of 118362 Lys residues, D) Distribution of 107556 Arg residues, E) Distribution of 26066 Cys residues.

Each type of amino acid in the dataset was binned according to its solvent accessibility and secondary structure. This provided information about the abundance of the different amino acids in the different solvent shells and their secondary structure preferences (Fig. 3). In order to retrieve the data displayed in Figure 3 we have written the following files: Hssp caller.m, HsspRead.m, rotateticklabel.m, SAplot.m, SolventAcc.mat. These codes have been uploaded as supplementary information in Figure S2 as “Hssp caller.m”, “HsspRead.m”, “rotateticklabel.m”, “SAplot.m”, and “SolventAcc.mat”. Furthermore, a description on how to install and run the software used to retrieve the solvent accessibility data displayed in Figure 3 can be found in the uploaded file “Figure S2 SAplots_User instructions.doc”.

### ProExtract

ProExtract combined the data from .ent and .hssp files into MATLAB structures, which were saved in .mat format (a MATLAB data file). For each .ent file, the atoms' coordinates and chain information were loaded into ProExtract, while information on residue type, secondary structure and solvent accessibility (SA) was loaded from the corresponding .hssp file. Since many .ent and .hssp files were found to contain errors, ProExtract included a validation routine, where residues as a minimum were required to have information on the Cα and functional atoms coordinates (*vide infra*), residue type, secondary structure, solvent accessibility (SA) and chain length. Furthermore, .hssp entries were required to have information about which residue and chain they corresponded to in the .ent file, as numbering in .hssp and .ent files might differ. Residues that did not have all the required information were discarded, while the rest of the chain information was retained. Those that were accepted were added to the MATLAB structure file for that protein. As a result, a file for each protein was created containing combined information on atom coordinates and chains, residue types, secondary structure and SA.

As output, ProExtract created a database in the form of an 8D tensor from the .mat files. The tensor contained information about pairs of amino acids present in the different SA protein shells. Two amino acids were considered a pair if they belonged to the same chain, were within the same SA-bin and had a distance less than 8.25 Å between their functional atoms (*vide infra*). Each of the eight tensor directions was binned according to:

- Type of the first amino acid (AA1) (20 bins)
- Type of the second amino acid (AA2) (20 bins)
- Solvent accessibility of the amino acid pair (SA) (12 bins)
- Distance between atoms in functional groups (D) (14 bins)
- Secondary structure for the first amino acid (SS1) (4 bins)
- Secondary structure for the second amino acid (SS2) (4 bins)
- Chain length (CL) (12 bins)
- Sequence distance between AA1 and AA2 (SD) (6 bins)

A pair of amino acid residues is composed of two amino acid residues. The first dimension tells us what is the type of the first amino acid (AA1) out of 20 possible amino acid types and the second dimension tells us what is the type of the second amino acid (AA2) in a pair (once again out of 20 possible amino acid types). So, the two first dimensions give us information about the nature of each amino acid in an amino acid pair in the protein. The fifth dimension is simply telling us in which secondary structural element the first amino acid is located (α-helix, β-strand, turn or coil). This information is retrieved from the downloaded .hssp file associated to each .pdb file. The same is valid for the sixth dimension: it tells us in which secondary structural element the second amino acid in a pair is located (α-helix, β-strand, turn or coil). This information is also retrieved from the .hssp file.

See bin definitions in section “*Bin definitions and functional atoms*”. 8272 .mat files were processed successively. All possible combinations of two residues were carried out to test if the two residues would constitute a pair (*vide supra*). When a pair was identified, the count in the data tensor cell with the coordinates (AA1, AA2, SA, D, SS1, SS2, CL, SD) was increased by one. A total of 5.211.796 pairs were identified. These were distributed between 1.756.714 cells in the tensor.

In order to establish the significance of the coordinates of each of the pairs in a protein, ProExtract shuffled the amino acids in accordance to the amino acid distribution of the protein. This process was repeated 10 times for each of the 8272 proteins, and the resulting 8D tensors were averaged. The average 8D tensor was used as a reference dataset. For a given pair, the ratio between the actual count in a cell in the observed 8D tensor and the average count in the reference dataset was a measure of the significance of the cell. We compute the ratio between the actual findings and the randomized value – a value above 1 indicates statistical significance. In the ratio plots all displayed data has been normalized (values between 0 and 1).

As output, ProExtract created an index dataset which could be used to identify the specific interactions that gave rise to the counts in a tensor cell. The index set was an 8D MATLAB cell character array. Whenever a pair was registered, a string was added to the corresponding cell in the index array of the form “1ABC0102A1030B” for the imaginary pair of amino acids 102A and 1030B in Protein Data Bank (PDB) structure 1ABC. When more than one pair was registered in the same cell, a new line was created for each pair in the cell. In this way it was possible to retrieve the protein(s) as well as the local fold context around an amino acid pair that contributed to the count in a particular cell.

Completing both the 8D tensor for the observed pairs, the 10 times averaged reference dataset as well as the index array took approximately one week of computational time on one processor in a Lenovo Thinkpad T500 with an Intel Core 2 Duo P8600 CPU at 2.4 GHz and with 4 GB RAM, running 64-bit MATLAB.

### Bin definitions and functional atoms

The first dimension of the dataset tensor had 20 amino acid bins: Ala, Arg, Asn, Asp, Cys, Gln, Glu, Gly, His, Ile, Leu, Lys, Met, Phe, Pro, Ser, Thr, Trp, Tyr, Val. The second dimension had 20 amino acid bins, identical to the first dimension. The third dimension had 12 solvent accessibility bins (SA in %): SA≤0, 0<SA≤10, 10<SA≤20, 20<SA≤30, 30<SA≤40, 40<SA≤50, 50<SA≤60, 60<SA≤70, 70<SA≤80, 80<SA≤90, 90<SA≤100, SA>100. The fourth dimension had 14 distance bins (D in Å): D≤1.75, 1.75<D≤2.25, 2.25<D≤2.75, 2.75<D≤3.25, 3.25<D≤3.75, 3.75<D≤4.25, 4.25<D≤4.75, 4.75<D≤5.25, 5.25<D≤5.75, 5.75<D≤6.25, 6.25<D≤6.75, 6.75<D≤7.25, 7.25<D≤7.75, 7.75<D≤8.25. The fifth dimension had four secondary structure bins for AA1: α-helix, β-strand, turn and coil. The sixth dimension had four secondary structure bins for AA2, identical to the fifth dimension. The seventh dimension had 12 chain length bins: CL≤0, 0<CL≤100, 100<CL≤200, 200<CL≤300, 300<CL≤400, 400<CL≤500, 500<CL≤600, 600<CL≤700, 700<CL≤800, 800<CL≤900, 900<CL≤1000, CL>1000. The eighth dimension had 6 sequence distance bins: 0, 1, 2, 3, 4, >4.

The functional atoms were for Ala CB, Arg NH1 and NH2, Asn ND2 and OD1, Asp OD1 and OD2, Cys SG, Gln NE2 and OE1, Glu OE1 and OE2, Gly CA, His ND1, Ile CG1 and CG2, Leu CG, Lys NZ, Met SD, Phe CZ, Pro CG, Ser OG, Thr OG1, Trp CE2, Tyr OH, Val CG1 and CG2 (atom nomenclature as described in the .ent files).

### ProPack

ProPack is a query program that allows the user to access the 8 dimensional data tensor. After loading the tensors created by ProExtract, two tensors were available for the program, one based on the observed data and one from the randomized reference data. The user could then request access to the observed data (‘absolute mode’), the randomized data (‘reference mode’) or the ratio between the observed and the randomized data (‘ratio mode’). Finally, the user could select a so called ‘warp mode’, where the absolute data was displayed as a 3D topographic map colored according to the intensity of the ratio data. Red color codes for highly significant data, while dark blue codes for less significant data.

The 8D tensor could be queried with any set of parameters. All dimensions that we are not querying are projected onto the subspace that we are visualizing. Therefore, if we intend to produce a 2D plot of the spatial distance (D) *vs* solvent accessibility (SA) of a specific amino acid pair e.g. Lys-Asp, we project the 4 remaining dimensions (SS1, SS2, CL, SD) onto the D-SA subspace.

ProPack contains a multi dimensional query language that allows the user to pose more elaborate and specific questions: e.g. we could ask for a 2D plot of cysteine residues specifically located in coil segments. Our 2D plot could be *distance vs. sequence distance* between the two cysteine residues. The user could also query the structural preferences for sets of amino acids, such as Arg-Lys and the Asp-Glu pairs.

### ProPair

ProPair is a query program that allows the user to select a particular set of cells in the data tensor. ProPair retrieved a list of proteins identified by the index dataset (*vide supra*) with pairs that corresponded to the given parameters. These pairs were presented to the user, with the possibility of being visualized using the Molviewer functionality in MATLAB. When interesting features had been located in the ProPack plots, it was thus possible to use ProPair to “go back to the source” and identify which amino acid pairs in which proteins contributed to those features.

## Results

The resolution of the protein structures used in this study is displayed in Figure 1A. In Figure 1B is depicted a spherical model of a globular protein displaying the solvent accessible (SA) shell concept. The protein residues are binned in solvent shells ranging from totally buried (0% SA) to full solvent exposure in steps of 10%, with 0% and >100% treated explicitly.

### Amino acid residues distribution (Asp, Glu, Lys, Arg, Cys)

In Figure 3 is displayed the solvent accessibility distribution for each amino acid residue together with the preference for being located in a particular secondary structural element (α-helix, β-strand, turn and coil). All amino acids will be mentioned using their 3 letter code. The distribution plots of the remaining amino acid residues can be found as supplementary information (Figure S2). In Figure 3A it can be seen that Asp prefers to be located in protein shells displaying solvent accessibility >0% up until ∼60%. Its distribution peaks in the solvent shells displaying solvent accessibility >0% and ≤10% and with solvent accessibility >40 and ≤50%. Asp is rarely present in the completely buried core of the protein (0% SA). In solvent shells with SA beyond 60%, the propensity for Asp declines linearly with increasing SA. In Figure 3B it can be seen that the SA distribution of Glu is similar to the Asp distribution, although Glu is less frequent than Asp in shells with SA≤20%. Glu is most frequently observed in the protein shell with SA between 50 and 60%. Interestingly, both Asp and Glu do not like to be fully solvent accessible.

Figure 3C shows that the distribution of Lys displays a Gaussian profile, peaking in the 40–50% SA shell, and falls off rapidly both with increasing and decreasing SA. Interestingly, the distribution of Arg is similar to the distribution of the oppositely charged Asp residue, preferring to be located in protein shells displaying solvent accessibility >0% and ≤60%. All four titratable residues avoid being located in the totally buried core (0% SA) of the protein, as well as in the highly solvent accessible shells. On the other hand, Cys residues prefer to be buried in proteins. Its distribution peaks in shells with SA≤10%, decaying afterwards exponentially towards increasing SA shells. Cys is almost completely absent in shells with SA>80%.

The secondary structural preferences for Asp, Glu, Lys, Arg and Cys are also displayed in Figure 3. Asp displays a preference for being located in coil structures in all solvent shells. Glu prefers to be located in α-helices whereas Lys and Arg display a preference for both α-helix and coil structures with a slight preference for the former. In general, for Glu, Lys and Arg the fractions of residues in coil and turn increase with solvent accessibility. For all four residues, the fraction of residues in β-strands decreases in an exponential way in protein shells with SA>50–60%. Cys prefers to be located both in α-helix or β-strands if completely buried (0% SA shell). In the protein shell with SA between 0–10%, Cys prefers to be located in coil structures, followed by a similar preference for α-helix and β-strands. When presence in shell with SA>10%, Cys prefers to be located in coil structures.

### Cysteine residues' interactions

**Figure 4:**
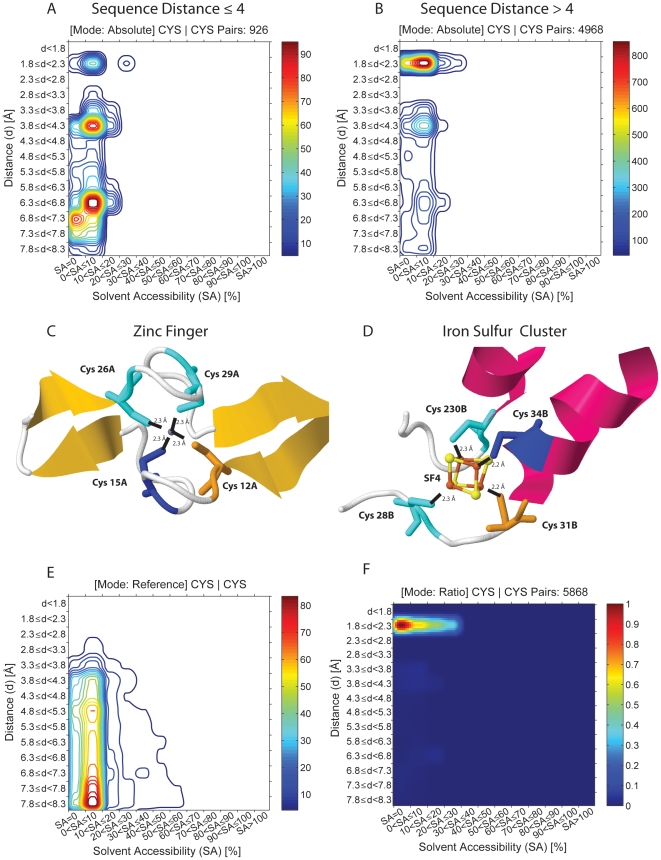
Distribution of the observed spatial distance (Å) and solvent accessibility of the protein shell where 923 Cys-Cys pairs located at a sequence distance ≤4 residues (4A) and at a sequence distance >4 residues (4B) are found. The intensity map is color coded (blue to red) and next to the color bar is displayed the number of pairs corresponding to each color code. Protein structures containing Cys-Cys pairs representative of the two major peaks displayed in 4A have been retrieved with the ProPair program and are displayed in 4C and 4D. Figure 4C shows that Cys-Cys pairs with distances peaking between 3.8–4.3 Å are part of a classical zinc finger motif in proteins. The Cys-Cys pair is displayed in dark blue and yellow. Two other Cys residues are displayed in cyan. Zn is displayed as a blue sphere. Figure 4D shows that Cys-Cys pairs with distances peaking between 6.3–7.3 Å are part of yet another classical cluster, the iron sulfur cluster. The Cys-Cys pair is displayed in dark blue and yellow. In the Fe_4_S_4_ cluster, Fe is displayed in orange and S in yellow. Figure 4E shows the distribution of the observed spatial distance (Å) and solvent accessibility of the protein shell where 923 Cys-Cys pairs located at a sequence distance >4 residues are found in a randomized reference database (see Methods section). Figure 4F was obtained by dividing the absolute data for Cys-Cys pairs with a sequence separation larger than 4 residues (Fig. 4B) by the reference dataset data (Fig. 4E), this way displaying the statistically relevant peaks.

Figure 4 displays the occurrence of Cys-Cys pairs as a function of spatial distance between the two cysteine residues forming a pair and the solvent accessibility of the protein shell where the pair is found. In Figure 4A the sequence distance between the Cys residues is equal to or less than 4 residues while in Figure 4B the sequence distance is larger than 4. For sequence distances ≤4, 926 pairs are found. The vast majority are found below 20% SA. The highest occurrence of pairs is seen for spatial distances between the two cysteines of 3.8–4.3 Å and 6.3–7.3 Å. Those pairs are located in a protein shell with SA≤10%. Furthermore, Cys-Cys pairs are also observed at spatial distances of 1.8–2.3 Å, though less frequently. When observing Figure 4B we can see that 4968 Cys-Cys pairs are found. These pairs are also located in protein shells with SA≤10% but the preferred spatial distance between these cysteine pairs is 1.8–2.3 Å. Fewer pairs are observed at spatial distances of 3.8–4.3 Å and 6.3–7.3 Å. Protein structures containing Cys-Cys pairs representative of the two major peaks displayed in Figure 4A (sequence distance less than 4) have been retrieved with the ProPair program and are displayed in Figures 4C and 4D. Figure 4C shows that Cys-Cys pairs with distances peaking between 3.8–4.3 Å are part of a classical zinc finger motif in proteins. ProPair shows that the majority of the hits are zinc fingers. The local structure around the zinc finger in 2bx9.pdb, a protein involved in transcription regulation, is displayed. This protein has 12 cysteine-rich zinc-binding domains. Figure 4D shows that Cys-Cys pairs with distances peaking between 6.3–7.3 Å, are part of yet another classical cluster: the iron sulfur cluster. The ProPair program shows that the majority of the Cys pairs with distances peaking between 6.3–7.3 Å are found in iron sulfur clusters. ProPair also finds that Cys residues with large sequence separation but spatially close to each other (peak at 1.8–2.3 Å in Fig. 4B) are involved in disulphide bridges.

In order to judge the uniqueness of the results displayed in Figure 4B, data has been compared with the Cys-Cys pair occurrences observed when using a dataset of randomized structures. It can be observed in Figure 4E that nearly all Cys-Cys pairs found in the randomized reference dataset are located in protein shells with 0<SA≤10%. The majority of the pairs have inter-residue distances between 6.8 to 8.3 Å. Pair occurrence decreases with decreasing distances. Almost no pairs are observed below ∼3.8 Å. The data displayed in Figure 4F was obtained by dividing the absolute data for Cys-Cys pairs with a sequence separation larger than 4 residues (Figure 4B) by the reference dataset data (Fig. 4E). It can be seen that the Cys-Cys pairs cluster in protein shells with 0–30% SA and that their distances lie between 1.8–2.3 Å as displayed (Fig. 4F). These preferences are absent in the randomized dataset (Fig. 4E).

**Figure 5:**
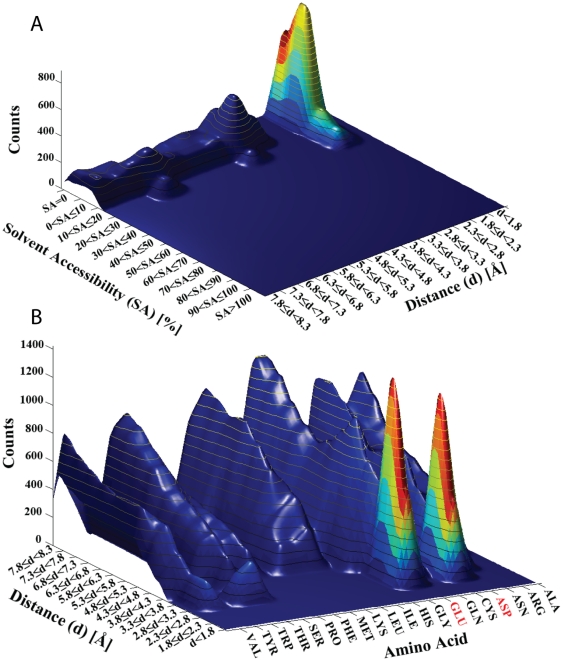
The relevance of warp plots. A) Warp plot merging information about the absolute number of pairs observed in Figure 4B with information about the ratio information displayed in Figure 4F, showing the statistical relevance of each peak. B) Warp plot showing the statistically relevant pairwise interactions that Lys residues make with other amino acids residues and at which spatial distances those interactions occur. The intensity map is color coded, dark blue coding for low statistic relevance and red for the highest statistic relevance.

In Figure 5A is displayed a warp plot which merges information about the absolute number of pairs observed in Figure 4B with information about the ratio information displayed in Figure 4F. The significant red peak (1.8–2.3 Å) that was observed in Figure 4F is seen to coincide with a high pair concentration at this location (indicated by the topography). This provides a statistical fundament of the observation. It can also be observed that the number of pairs found at distances of 3.8–4.3 Å and 0–10% SA is significant but that these pairs do not represent unique preferences (dark blue color).

### Interactions between acid and basic residues

In Figure 5B is displayed a warp plot showing the statistically relevant pairwise interactions that Lys residues make with other amino acids residues and at which spatial distances those interactions occur. It can be observed that Glu and Asp residues are the closest preferred neighbors seen at distances peaking around 2.3–3.3 Å (red peaks). All other observed dark blue peaks are less statistically relevant interactions.

### Pairs between residues with opposite charge

**Figure 6:**
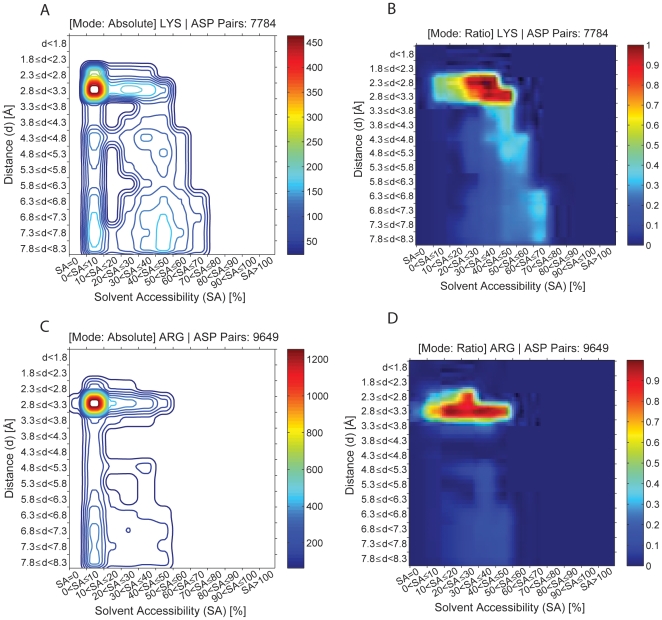
Occurrence of pairs between opposite charge residues as a function of the spatial distance between them and the solvent accessibility of the shell where the pair is found. The so called “Absolute” plots display the number of contacts found and the “Ratio” plots are the ratio between the data in the absolute plots and the corresponding data found in the reference dataset of randomized structures. Figures 6A and 6B report the contacts found between 7784 Lys-Asp pairs. Figures 6C and 6D report the contacts found between 9649 Arg-Asp pairs. The intensity map is color coded like described in Figure 4.

In Figure 6 is displayed the occurrence of pairs between residues with opposite charge as a function of the spatial distance between them and the solvent accessibility of the shell where the pair is found. The so called “Absolute” plots display the number of contacts found and the “Ratio” plots are the ratio between the data in the absolute plots and the corresponding data found in the reference dataset of randomized structures. Ratio plots are color coded: red codes for statistically relevant peak and dark blue for a statistically non-relevant peak. Figures 6A and 6B report the contacts found between 7784 Lys-Asp pairs. Figures 6C and 6D report the contacts found between 9649 Arg-Asp pairs. 9186 Lys-Glu pairs and 11944 Arg-Glu pairs were found (data present in Figure S3). Both absolute and ratio plots show that pairs of opposite charge are preferentially found at close distances between 2.3–3.3 Å allowing for salt bridge formation and in protein shells with 0<SA≤50%.

### Pairs between residues with the same charge

**Figure 7:**
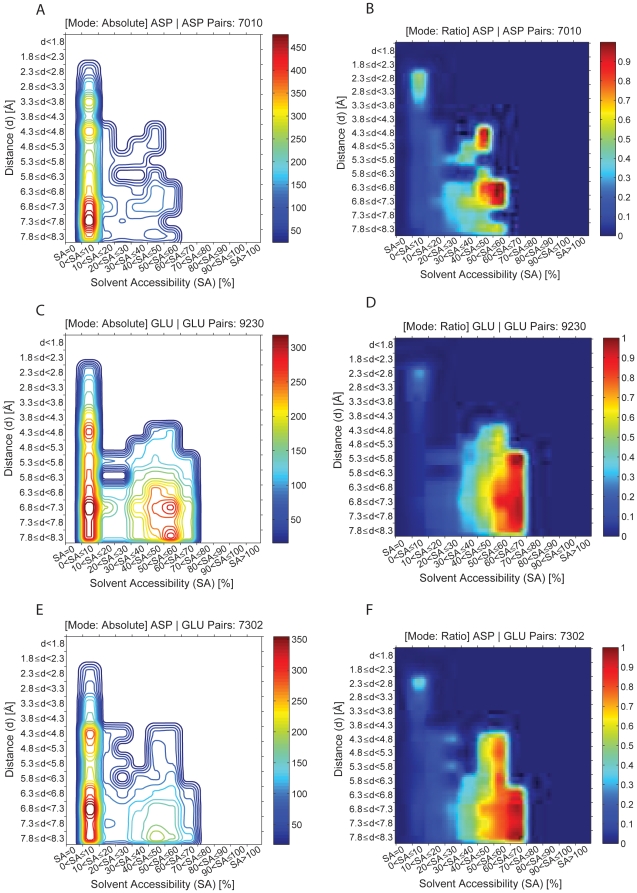
Occurrence of pairs between negative charge residues as a function of distance between any two residues and the solvent accessibility of the shell where the pair is found. As explained in Figure 6, absolute (panels A, C and E) and ratio (panels B, D and F) plots are displayed for each pair. A total of 7010 Asp-Asp pair (panels A and B), 9230 Glu-Glu pairs (panels C and D), 7302 Asp-Glu pairs (panels E and F) were analyzed. The intensity map is color coded like described in Figure 4.

**Figure 8:**
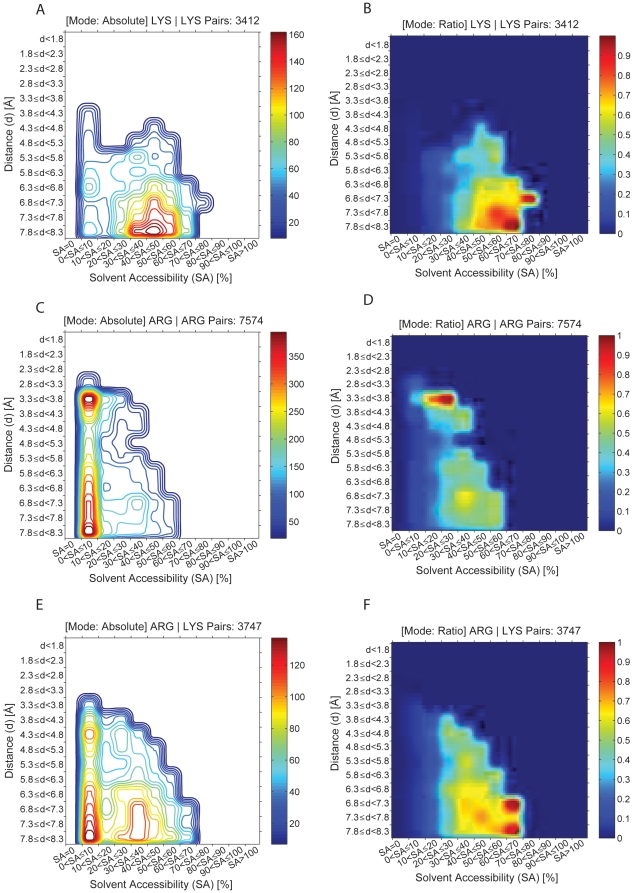
Occurrence of pairs between positive charge residues as a function of distance between any two residues and the solvent accessibility of the shell where the pair is found. As explained in Figure 6, absolute (panels A, C and E) and ratio (panels B, D and F) plots are displayed for each pair. A total of 3412 Lys-Lys pairs (panels A and B), 7574 Arg-Arg pairs (panels C and D), 3747 Arg-Lys pairs (panels E and F) were analyzed. The intensity map is color coded like described in Figure 4.

In Figures 7 and 8 are displayed the occurrences of pairs between same charge residues as a function of distance between any two charged residues and the solvent accessibility of the shell where the pair is found. As explained above, absolute and ratio plots are displayed for each pair. A total of 7010 Asp-Asp pairs, 9230 Glu-Glu pairs, 7302 Asp-Glu pairs, 3412 Lys-Lys pairs, 7574 Arg-Arg pairs, 3747 Arg-Lys pairs were analyzed. The ratio plots show that residues of the same charge prefer to be located further away from each other (pairs rarely seen at distances below 4.3 Å) and in solvent layers with higher SA (40<SA≤80%) when compared to oppositely charged residues (Figs. 7B, 7D, 7F, 8B and 8F), except for Arg-Arg pairs which prefer to be located in protein shells with 10<SA≤30% at short spatial distances between 3.3–3.8 Å (Fig. 8D, details below).

### Asp-Asp, Glu-Glu and Asp-Glu preferences

The absolute and ratio plots do not necessarily provide the same information. Figure 7A shows that the majority of Asp-Asp pairs are found in 0<SA≤10% shells peaking at the preferred distance of 6.8–7.8 Å, with minor peaks at 4.3–4.8 Å and 3.3–3.8 Å. Figure 7B on the other hand shows that the preferred spatial distances between residues in the majority of the statistically relevant pairs peak at 6.3–7.3 Å and 4.3–5.3 Å, and that these pairs are preferentially seen at 40–60% SA. An interesting unexpected peak is observed at 2.3–3.3 Å and 0<SA≤10%.

Figure 7C shows that Glu-Glu pairs are found preferentially at 0<SA≤10% and 40<SA≤60%. However, Figure 7D shows that only the pairs found at 40<SA≤70% are different from the reference set of randomized structures and therefore statistically relevant. Figure 7D also shows that the preferred distances between the Glu-Glu residues are 5.3–8.3 Å. Figure 7E shows that the Asp-Glu pairs are preferentially found at 0<SA≤10% with preferred distances at 4.3–4.8 Å and 6.3–8.3 Å. However, Figure 7F shows that only the pairs found at 40<SA≤70% are statistically relevant and that the preferred distances between the Asp-Glu residues are observed between 4.3–8.3 Å. Distances below 4.3 Å are allowed in those protein shells. Both for Glu-Glu and Asp-Glu pairs an unexpected peak is observed at 2.3–2.8 Å and 0<SA≤10%, as observed for Asp-Asp contacts.

### Lys-Lys, Arg-Arg and Arg-Lys preferences

The Lys-Lys pair preferences are quite different from the Arg-Arg pair preferences, despite both residues being positively charged. Figure 8A shows that Lys-Lys pairs are preferentially observed at a spatial distance above 6.8 Å in protein shells with 30<SA≤60%. Figure 8B shows that the statistically relevant contacts appear at distances above 6.3 Å and 40<SA≤80%. Figure 8C shows that the Arg-Arg pairs are preferentially observed at low SA shells (0<SA≤10%) and the preferred distances are 3.3–3.8 Å and above 6.3 Å. Figure 8D shows that the only statistically relevant peaks occur at 10<SA≤30% with a distance between residues of 3.3–3.8 Å.

Figure 8E shows that Arg-Lys pairs are seen in a wide range of SA, preferentially from 0<SA≤50% with preferred inter-residue distances above 6.3 Å and between 4.3–4.8 Å. Figure 8F shows that only the pairs found at 30<SA≤70% are statistically relevant, especially the pairs found at 60<SA≤70%. In these preferred protein shells, Arg-Lys pairs are observed at distances above 6.8 Å.

### Secondary structural preferences of pairs of residues

**Figure 9:**
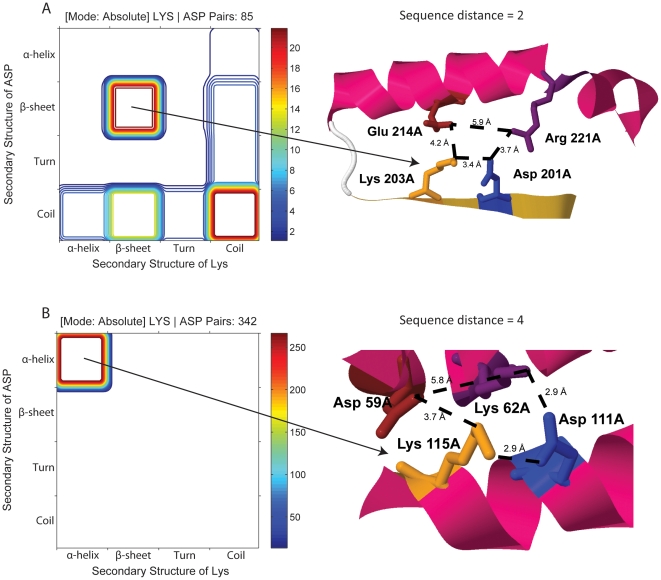
Pair preferences for particular secondary structural elements. A) Secondary structural elements preferred by Lys and Asp pairs with a sequence distance of 2 and with inter-residue distances between 2.3–3.8 Å in protein shells with 20<SA≤40%. Lys and Asp prefer to be located in β-sheets or in coil structures. A typical local 3D structure around a Lys-Asp pair (displayed in yellow and blue, respectively) with the characteristics depicted by the beta-beta peak is displayed. Distances between functional groups are displayed. B) Secondary structural elements preferred by Lys and Asp pairs with a sequence distance of 4 and with inter-residue distances between 2.3–3.8 Å in protein shells with 20<SA≤40%. Lys and Asp (displayed in yellow and blue, respectively) prefers to be located exclusively in α-helices. A larger salt bridge network involving two additional residues (Asp 59 and Lys 62, displayed in brown and purple, respectively) located in a nearby α-helix is displayed.

As mentioned above, the ratio plot of Lys-Asp reveals that the statistically relevant pairs are found between 2.3–3.3 Å and in protein shells with 10<SA≤50% (Fig. 6B). In Figure 9A is displayed the secondary structural elements preferred by Lys and Asp pairs with a sequence distance of 2 and with inter-residue distances between 2.3–3.8 Å in protein shells with 20<SA≤40%. A total of 85 pairs met the criteria. It can be seen that those Lys and Asp prefer to be located in β-sheets or in coil structures. In Figure 9A is also displayed a typical local 3D structure around a Lys-Asp pair with the characteristics depicted by the beta-beta peak: it can be seen that the Lys-Asp pair (Lys203-Asp201 displayed in yellow and blue) forms a salt bridge (3.4 Å distance between functional charged groups) and is part of a β-strand. This salt bridged pair is involved in a larger salt bridge network involving two additional residues (Glu 214 and Arg 221, displayed in brown and purple, respectively) located in a nearby α-helix. The distances between these residues allow for additional salt bridge formation. The depicted protein (1SC3.pdb) is a human caspase (interleukin-1 beta convertase), an enzyme that proteolytically cleaves the precursor form of the inflammatory cytokine interleukin 1-β into its active mature peptide.

In Figure 9B the corresponding plot is displayed for a sequence distance of 4 between Lys and Asp. 342 pairs from the dataset met the selection criteria and were analyzed. It can be seen that Lys and Asp now prefer to be located exclusively in α-helices, forming a salt bridge that stabilizes one turn of the helix. The functional groups of Lys 115 and Asp 111 (displayed in yellow and blue, numbered according to 3CLJ.pdb) are within 2.8 Å, suggesting a strong salt bridge. Furthermore, these residues are involved in a larger salt bridge network involving two additional residues (Asp 59 and Lys 62, displayed in brown and purple) located in a nearby α-helix. The distances between these residues allow for additional salt bridge formation (Fig. 9B). 3CLJ is an α-helical RNA polymerase II.

**Figure 10:**
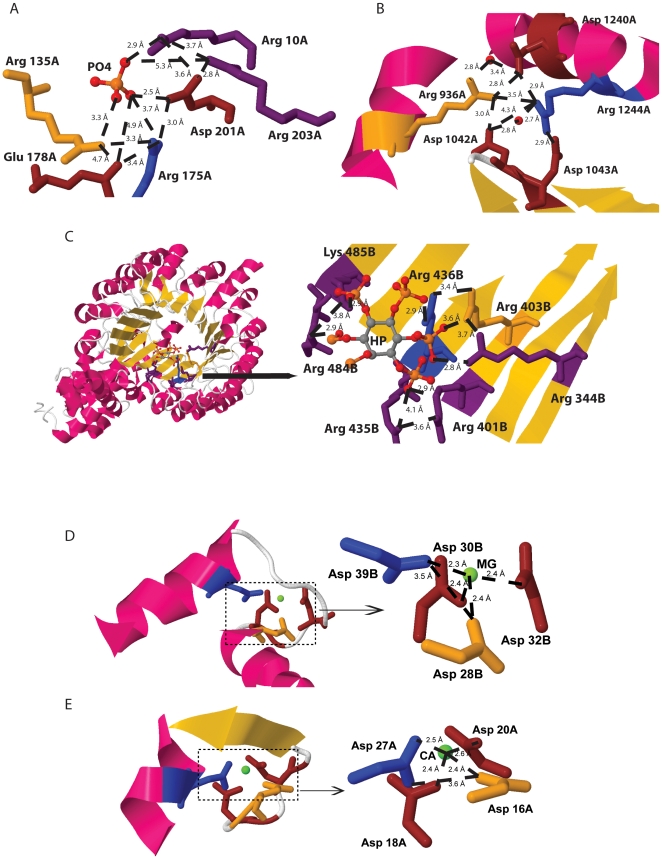
Figures 10A to 10C display typical protein motifs involving Arg-Arg pairs that are in close proximity (3.3–3.8 Å) in protein shells with 10–30%SA. Arg residues should be within a sequence distance larger than 4 and located in coil elements (Fig. 10A, PO_4_ is a phosphate group, 2HNH.pdb), in α-helix (Fig. 10B, 202K.pdb, structural water molecules are displayed as red spheres) or in β-strands (Fig. 10C, 2P1M .pdb, HP is a sugar molecular linked to six phosphate group). Figures 10D and 10E displayed typical protein motifs involving Asp-Asp pairs that are in close proximity (2.3–3.3 Å) in protein shells with 0<SA≤10% (1WDC.pdb and 2SCP.pdb, respectively). Such Asp-Asp pairs (displayed in blue and yellow) are involved in Mg^2+^ and Ca^2+^ binding involving 2 additional Asp residues (displayed in brown). Distances between functional groups are shown.

In Figures 10A to 10C are displayed typical protein motifs involving Arg-Arg pairs that are in close proximity (3.3–3.8 Å) in protein shells with 10–30%SA. These were the characteristics of the statistically most relevant and unexpected peak displayed in Figure 8D. Further parameters were used when searching for structures containing these pairs: Arg residues should be within a sequence distance larger than 4 and could be located in coil elements (Fig. 10A), in α-helices (Fig. 10B) or in β-strands (Fig. 10C). Figure 10A shows that the Arg-Arg pair can bind a phosphate group and at the same time be involved in a multiple salt bridge network. The distances between functional groups are displayed. The protein (2HNH.pdb) is the catalytic alpha subunit of the *E. coli* replicative DNA polymerase III. Figure 10B shows that nearby Arg-Arg pair (Arg936-Arg1244 displayed in yellow and blue, respectively) present in helical elements can be involved in a multiple salt bridge network involving 3 titratable residues (Asp1042, Asp1043 and Asp1240 displayed in brown) The closest distances between the functional groups of these residues (from 2.8 Å to 4.3 Å) allow for salt bridge formation. The depicted protein is 202K.pdb. Figure 10C shows how nearby Arg-Arg pairs present in beta strands can be involved in multiple salt bridge network involving 7 titratable residues (Lys and Arg residues) present in six adjacent β-strands and 6 phosphate groups. The pair in question is Arg403-Arg436 (3.4 Å apart, colored yellow and blue) present in 2P1M.pdb, an outer membrane protein involved in lipid deacylation. The closest distances (2.9–4.1 Å) to the functional groups of other Lys and Arg residues (displayed in purple) are displayed.

In Figures 10D and 10E are displayed typical protein motifs involving Asp-Asp pairs that are in close proximity (2.3–3.3 Å) in protein shells with 0<SA≤10%. These were the characteristics of the unexpected peak displayed in Figure 7B. Such Asp-Asp pairs (displayed in blue and yellow) are involved in Mg^2+^ and Ca^2+^ binding involving 2 additional Asp residues (displayed in brown). The pair Asp28-Asp39 displayed in Figure 10D belongs to 1WDC.pdb, a myosin regulatory domain of a muscle protein. The residues are located in two nearby α-helices while the two additional Asp residues are located in loop regions. The distances between the charged functional groups of each Asp residue to Mg^2+^ are displayed in Figure 10D. The pair Asp16-Asp27 displayed in Figure 10E (displayed in yellow and blue. 3.6 Å apart) belongs to 2SCP.pdb, a sarcoplasmic calcium-binding protein. Asp16 is located in a loop region and Asp27 is located in an α-helix. Two additional Asp residues are also involved in Ca^2+^ binding (Asp18 and Asp20, colored brown).

## Discussion

In the introduction we have argued for a total of 8 dimensions spanning the protein fold space. These 8 dimensions may be orthogonal to one another, or each dimension may be linear combinations of two or more of the other dimensions. For example the sequence length will define the size of the protein and thereby also influence the number of both solvent exposed and buried residues. No matter of how many independent dimensions we can define, only two dimensions are usually displayed simultaneously. A third dimension can be added in terms of a color. The bioinformatics approach presented in this paper allows the user to carry out hyper dimensional analyses of amino acid pair interactions and their distribution in proteins with an incorporated graphical analysis tools, making possible the visualization of any conceivable combination of the 8 dimensions for each amino acid pair as projections in 1D, 2D and 3D plots. The data cube obtained by ProExtract can be queried using the programs ProPack and ProPair. We have presented several arguments for perceiving the protein fold as spanned by at least 8 dimensions. In an upcoming paper we will show that the individual filled cells of the 8 dimensional fold tensor form a nearly perfect scale free organization. The hyper-dimensionality of our approach allows, e.g., specifying the precise SA of amino acid pair, instead of simply classifying the pair as buried or exposed. However, the present approach only considers amino acid pairs if they share the same solvent accessibility shell. Clearly one can consider amino acid pairs that bridge neighboring solvent shells as well. In the present context we imposed this restriction in order to limit the complexity of our dataset.

Different motifs involving cysteine residues such as disulphide bridges, zinc fingers and iron-sulfur clusters are clearly identifiable and differentiated with ProPack and confirmed by ProPair when 5 out of 8 constraints are imposed: AA1, AA2, solvent accessibility, spatial and sequence distances between the pair of cysteines residues (Figs. 4A, 4B, 4C and 4D). Amino acid pairs present in zinc fingers and sulfur iron clusters are seen in immediate sequence vicinity (sequence distance ≤4). In the zinc fingers those pairs have a preferred spatial distance between 3.8–4.3 Å (Figs. 4A and 4C) while pairs present in sulfur iron clusters have a preferred spatial distance between 6.3–7.3 Å (Figs. 4A and 4D). Disulphide bridges are clearly seen as longer range interactions (sequence distance >4) than zinc fingers and sulfur iron clusters. The preferred distance between the sulfur atoms is found by ProPack to be between 1.8–2.3 Å. Both observations are in agreement with previously published studies [41]. The results illustrate the potential of imposing constraints when querying the data cube.

The relevance of warp plots made by ProPack can be seen in Figure 5: merging of information about the absolute number of pairs with information about the ratio information leads to insight into the statistic relevance of each peak. An intense peak in the absolute mode plot is not necessarily statistically relevant, as shown in Figure 5B where the statistical relevance of each peak is color coded (red codes for the highest statistical relevance and dark blue for the lowest statistical relevance).

In addition to motifs involving cysteine residues, salt bridges were also investigated using ProPack and ProPair. Both absolute and ratio plots made by ProPack when 4 out of 8 constraints are imposed (AA1, AA1, solvent accessibility of the protein shell where the pair is located and spatial distance between the amino acid residues) show that pairs of oppositely charged amino acid residues are preferentially found at close distances between 2.3–3.3 Å allowing for salt bridge formation and in protein shells with 0<SA≤50% (Fig. 6). Our criterion for the existence of a salt bridge is the same as originally proposed by Barlow and Thornton [44], which is that the distance between the heavy atoms of the ionizable groups of the charged residues is <4 Å. Sarakatsannis and Duan [45] report that salt bridges display preferential formation in an environment of about 30% solvent accessibility surface area. Our data shows that Lys-Asp and Arg-Asp (Figs. 6B and 6D) pairs are observed to preferentially form salt bridges in protein shells with 20<SA≤50% and 0<SA≤50%, respectively. For Lys-Asp a peak is observed at 30–40% SA and for Arg-Asp two peaks are observed at 10–20% and 30–40% SA. Lys-Glu and Arg-Glu pairs (data shown in supplementary information) are observed to form salt bridges in protein shells with 20<SA≤60% and 10<SA≤50%, respectively. For both Lys-Glu and Arg-Glu a peak is observed at 30–40% SA. Furthermore, our programs also report the spatial distance observed between the functional groups of the residues involved in a salt bridge, as well its statistical relevance. Our data shows that few salt bridges are observed at protein shells with SA above 50–70%, depending on the particular amino acids involved in the salt bridge. Our results agree with the observation that no salt bridges were found in an environment with solvent accessibility surface area above 70% [45]. In addition, our data permits to specify the SA limit for each of the four salt bridges.

Figure 7 clearly shows the importance of displaying ratio plots of pair interactions (Figs. 7B, D and F) instead of absolute plots (Figs. 7A, C and E). The ratio plots allow for the display of the statistically relevant interactions between pairs of amino acid residues as a function of the solvent accessibility of the protein layer where the pair is located and as a function of spatial distance between the amino acid residues forming a pair. ProPack indentifies that the majority of amino acid pairs between negatively charged residues (Asp-Asp, Glu-Glu and Asp-Glu) avoid being in close spatial proximity (d>4.3 Å) and are preferentially seen in protein shells with SA≥40–50% (high intensity peaks displayed in Figs.7 B, D and F). This is expected since such larger spatial distances will prevent repulsion between residues carrying the same charge. However, ProPack also indentifies that Asp-Asp pairs in particular are seen in buried protein shells (0<SA≤10%) in unexpected close spatial proximity of 2.3–3.3 Å (Fig. 7B). ProPair allows the user to visualize these interactions in the structural location within a particular protein structure, as displayed in Figures 10D and 10E. Such Asp-Asp pairs are involved in Mg^2+^ and Ca^2+^ binding involving 2 additional Asp residues (displayed in brown). The pair Asp28-Asp39 displayed in Figure 10D belongs to 1WDC.pdb, a myosin regulatory domain of a muscle protein. This protein's enzymatic activity is switched on by direct Ca^2+^ binding. Mg^2+^ binds to the regulatory light chain of myosin. Mg^2+^ binds to a classical Ca^2+^ binding site (DXDXDG) containing the canonical helix-loop-helix structure, involving Asp28, Asp30, Asp32 and Asp 39. The Asp16-Asp27 pair displayed in Figure 10E belongs to a sarcoplasmic calcium-binding protein. Two additional Asp residues are also involved in Ca^2+^ binding (Asp18 and Asp20, colored brown). Together with Asp16 and Gly21 these residues are part of a classical DXDXDG Ca^2+^ binding sequence [62]. The above mentioned structural reasons justify the close spatial proximity between Asp-Asp pairs and their presence in buried protein shells. Both Ca^2+^ and Mg^2+^ binding are important for the activity of those proteins.

ProPack indentifies that pairs between positively charged residues (Lys-Lys and Arg-Lys) avoid being in close spatial proximity and are preferentially seen in protein shells with SA≥40–50% (high intensity peaks displayed Figs. 8B and F). This is expected since such larger spatial distances will prevent repulsion between residues carrying the same charge. However, ProPack also indentifies that Arg-Arg pairs are seen in buried protein shells (10<SA≤30%) in unexpected close spatial proximity of 3.3–3.8 Å (Fig. 8D). Our data opposes the pervasive belief that an Arg-Lys mutation is perceived as a conservative mutation. ProPair allows the user to visualize these interactions in the structural location within a particular protein structure, as displayed in Figures 10A, B and C. Further constraints were used when searching for structures containing these pairs: Arg residues should be within a sequence distance larger than 4 and could be located in coil elements (Fig. 10A), in α-helix (Fig. 10B) or in β-strands (Fig. 10C). Therefore the data displayed by ProPair in Figure 10 is a result of querying the data cube using a combination of 8 dimensions using the programs ProPack and ProPair. The 8 dimensions are: *AA1- Arg*, *AA2- Arg*, 10<SA≤30%, *D*- 3.3–3.8 Å, *SS1- coil*, *SS2- coil*, *CL*- all, *SD>4*. The displayed data shows the structural reasons that justify the presence of unexpected buried and spatially close Arg-Arg pairs: these positively charged pairs can bind a phosphate group and at the same time be involved in a complex salt bridge network with other titratable residues, this way contributing to the stability of the protein. Some of the salt bridges form so-called complex salt bridges, in which one charged residue forms salt bridges with two or more residues simultaneously [45]–[47], as displayed in Figures 10A and B. The energetic contribution of complex salt bridges has been suggested to have importance for protein stability. Gvritishvili et al. [48] showed that in two out of three cases, complex salt bridge formation is cooperative, i.e., the net strength of the complex salt bridge is more than the sum of the energies of individual pairs.

ProPair and Propack successfully identifies salt pair interactions in α-helices and in β-sheets when the data cube is queried using 5 constraints for a chosen amino acid pair. For example, in Figure 9A the 5 dimensions are: secondary structural element preferred by residue 1 (Lys), secondary structural element preferred by residue 2 (Asp), sequence distance of 2, inter-residue distances between 2.3–3.8 Å and protein shells with 20<SA≤40%. When these 5 constrains are imposed, ProPair allows the user to visualize these interactions in the structural location within a particular protein structure (Fig. 9). The typical local 3D structure around a Lys-Asp pair with the characteristics depicted by the beta-beta peak is displayed in Figure 9A. As known, two consecutive residues in a β-strand point in opposite directions. Therefore, the closest distance at which a salt bridge can be formed corresponds to residues with a sequence distance of 2. On the other hand, Figure 9B shows that if we impose that the Lys-Asp pair should have a sequence distance of 4, than ProPack identifies that this pair only seen as part of an α-helix. Indeed the closest distance at which a salt bridge can be formed in an α-helix corresponds to residues with a sequence distance of 4. These examples demonstrate that ProExtract, ProPack and ProPair correctly identify pair interactions between charged residues. Our results agree with the data published by Sarakatsannis and Duan [45], which shows that most α-helical salt bridges occurred with residue separation of 4, and the most frequent residue separation among β-sheets salt bridges is 2. We also observe that the most frequent residue separation among coils salt bridges is also 2 (Fig. 9A). The hyper-dimensionality of our bioinformatics approach allows imposing at the same time different constraints as described above. Together with ProPair data such as shown in Figure 9 can be retrieved. The approach by Sarakatsannis and Duan [45] allowed them to report the number of salt bridges *versus* secondary structure. Our approach allows us to specify at the same time that preference as a function of amino acid pair type (AA1 and AA2), solvent accessibility of the protein shell, spatial distance and residue sequence distance in a protein of a specified sequence length.

### Conclusions

The bioinformatics approach and results presented in this paper were only possible because the user is allowed to carry on hyper dimensional analyses of amino acid pair interactions in proteins. Furthermore, the incorporated graphical analysis tools enable the visualization of any conceivable combination of the 8 dimensions for each amino acid pair. The tools presented in this paper are likely to be of importance in the general field of protein engineering. Before considering creating a mutant protein for which the 3D structure is known, or can be predicted with reasonable accuracy, the hyperdimensional database can be queried if this substitution appears allowed or not. Considering that the query is done in a matter of minutes, this will always be faster than producing the mutant protein. The data cube established in the context of the present paper, is a representation of protein fold space. Thus all cells of the tensor with content different from 0 represent a particular feature that is allowed in protein folds. Pending on the tensor cell content, we may view this feature as more or less common. Conversely if a tensor cell is empty, it is likely to represent a particular feature that is disallowed in the protein fold space. Therefore our protein fold data cube could potentially be of value for efforts to predict or validate a protein fold.

Since user-friendly and publicly accessible web-servers represent the future direction for developing practically more useful models, simulated methods, or predictors [63], we shall make efforts in our future work to provide a web-server for the methods presented in this paper.

## Supporting Information

Figure S1 **Source codes and description on how to run the programs ProExtract, ProPack and ProPair.** The source code of the program ProExtract is “ProExtract_V2p4.m” with associated file “ProExtract_V2p4.fig” (located in the Figure S1 file). The source code of program ProPack is “ProPack.m” with associated file “ProPack.fig” (located in the Figure S1 file). The source codes of program ProPair are “PairFinder_v1p2.m”, with associated file “PairFinder_v1p2.fig”, and “PairSearcher_v1p1.m” (located in the Figure S1 file). A description on how to run the software ProExtract, ProPack and Propair can be found in files: “ProExtract_User instructions.doc”, “Propack_User instructions.doc, and “ProPair_user instructions.doc”, respectively. The two input files needed in order to run ProExtract are the protein.ent list and the list of correspondent hssp files. A file named “pisces_35_id_files_with_hssp.txt” contains the name of all pdb files that have been used. This file should be open with WordPad. The associated .ent and .hssp files are publically available.
(BZ2)

Figure S2 **SolventAccessibilityPlots - Histograms displaying the abundance of selected amino acid residues (Ala, Phe, Gly, His, Ile, Leu, Met, Asp, Pro, Glu, Ser, Thr, Val, Trp, Tyr) in each protein shell characterized by a specific solvent accessibility SA.** For each SA bin is also displayed the relative abundance of each selected residue in a particular secondary structural element (α-helix, β-strand, turn and coil). The first and last bins contain the fraction of residues in completely buried protein shell (0% SA) and in a completely solvent accessible protein shell (100% SA), respectively. The 3 letter amino acid residue is displayed in each distribution. The distribution has been obtained for 169461 Ala residues, 85634 Phe residues, 144453 Gly residues, 48403 His residues, 121232 Ile residues, 198265 Leu residues, 34636 Met residues, 85757 Asn residues, 94118 Pro residues, 76629 Gln residues, 120194 Ser residues, 110887 Thr residues, 147555 Val residues, 28894 Trp residues, 73340 Tyr residues.
(BZ2)

Figure S3 **ArgGlu Plots - Occurrence of ArgGlu pairs as a function of the spatial distance between the residues in each pair and the solvent accessibility of the shell where the pair is found.** The so called “Absolute” plots display the number of contacts found. The so called “Ratio” plots are the ratio between the data in the absolute plots and the corresponding data found in the reference dataset of randomized structures. The figures report the contacts found between 11944 ArgGlu pairs. The intensity map is color coded like described in Figure 4. **LysGlu Plots** - Occurrence of LysGlu pairs as a function of the spatial distance between the residues in the pair and the solvent accessibility of the shell where the pair is found. The so called “Absolute” plots display the number of contacts found. The so called “Ratio” plots are the ratio between the data in the absolute plots and the corresponding data found in the reference dataset of randomized structures. The figures report the contacts found between 9186 LysGlu pairs. The intensity map is color coded like described in Figure 4.
(PDF)
