# Supplementary material for: Hyperdimensional Analysis of Amino Acid Pair Distributions in Proteins
Source: PLoS One. 2011 Dec 9;6(12):e25638. doi: 10.1371/journal.pone.0025638 (PMC3235099; doi:10.1371/journal.pone.0025638)
Supplement: Figure S3 — ArgGlu Plots - Occurrence of ArgGlu pairs as a function of the spatial distance between the residues in each pair and the solvent accessibility of the shell where the pair is found. The so called “Absolute” plots display the number of contacts found. The so called “Ratio” plots are the ratio between the data in the absolute plots and the corresponding data found in the reference dataset of randomized structures. The figures report the contacts found between 11944 ArgGlu pairs. The intensity map is color coded like described in Figure 4. LysGlu Plots - Occurrence of LysGlu pairs as a function of the spatial distance between the residues in the pair and the solvent accessibility of the shell where the pair is found. The so called “Absolute” plots display the number of contacts found. The so called “Ratio” plots are the ratio between the data in the absolute plots and the corresponding data found in the reference dataset of randomized structures. The figures report the contacts found between 9186 LysGlu pairs. The intensity map is color coded like described in Figure 4. (PDF) [file pone.0025638.s003.pdf]

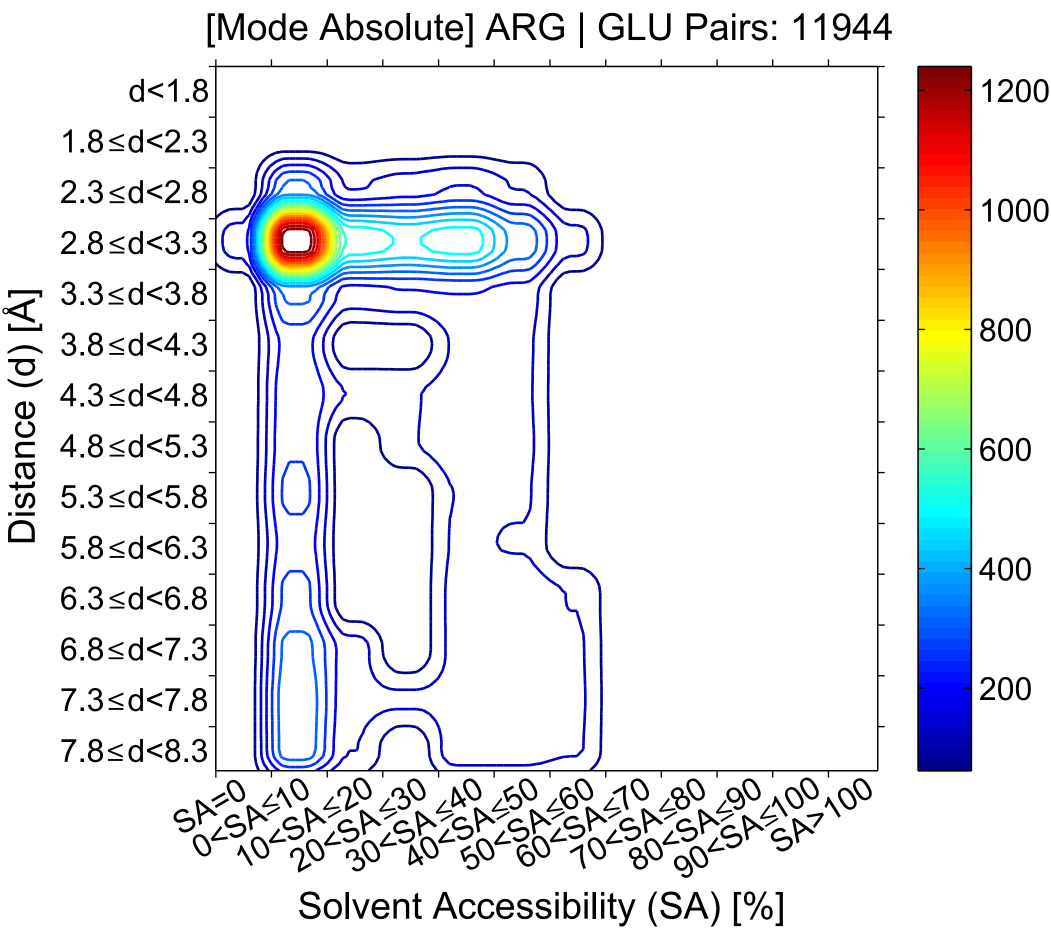
[Mode Absolute] ARG | GLU Pairs: 11944
Distance (d) [Å]
d<1.8
1.8≤d<2.3
2.3≤d<2.8
2.8≤d<3.3
3.3≤d<3.8
3.8≤d<4.3
4.3≤d<4.8
4.8≤d<5.3
5.3≤d<5.8
5.8≤d<6.3
6.3≤d<6.8
6.8≤d<7.3
7.3≤d<7.8
7.8≤d<8.3
SA=0
0<SA≤10
10<SA≤20
20<SA≤30
30<SA≤40
40<SA≤50
50<SA≤60
60<SA≤70
70<SA≤80
80<SA≤90
90<SA≤100
SA>100
Solvent Accessibility (SA) [%]
1200
1000
800
600
400
200

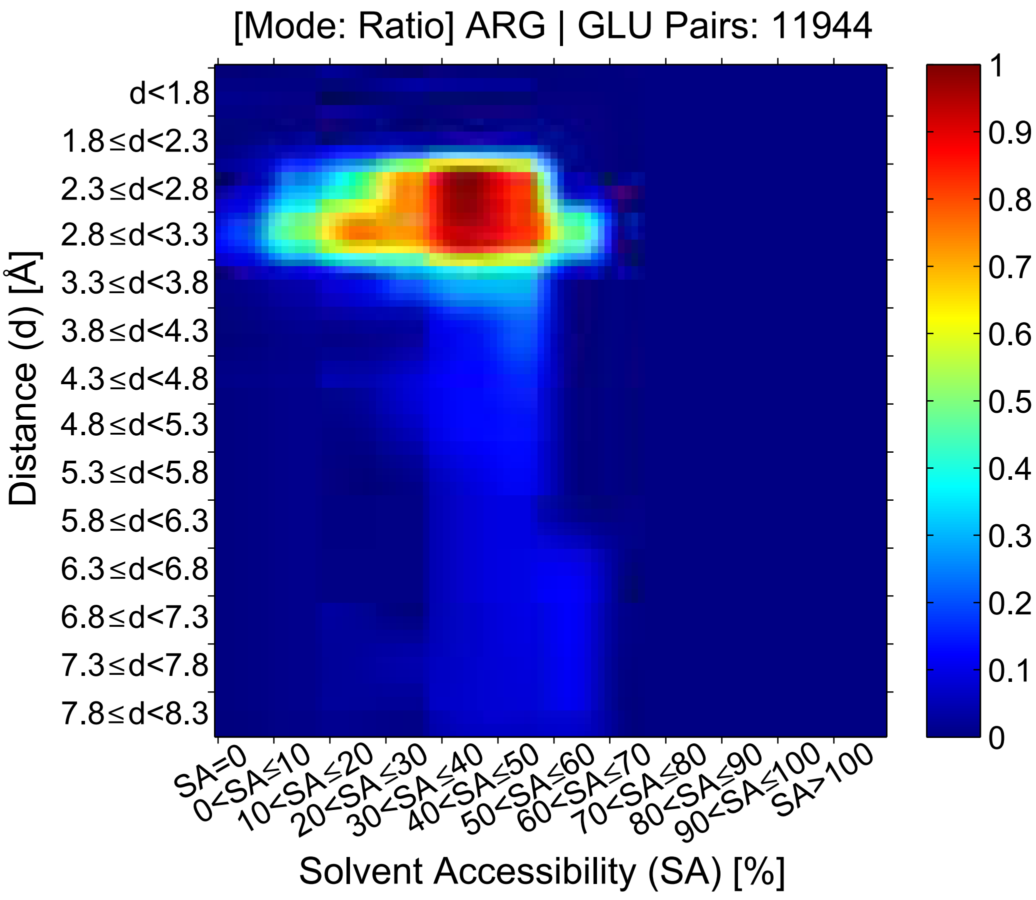
[Mode: Ratio] ARG | GLU Pairs: 11944
Distance (d) [Å]
d<1.8
1.8≤d<2.3
2.3≤d<2.8
2.8≤d<3.3
3.3≤d<3.8
3.8≤d<4.3
4.3≤d<4.8
4.8≤d<5.3
5.3≤d<5.8
5.8≤d<6.3
6.3≤d<6.8
6.8≤d<7.3
7.3≤d<7.8
7.8≤d<8.3
SA=0
0<SA≤10
10<SA≤20
20<SA≤30
30<SA≤40
40<SA≤50
50<SA≤60
60<SA≤70
70<SA≤80
80<SA≤90
90<SA≤100
SA>100
Solvent Accessibility (SA) [%]
1
0.9
0.8
0.7
0.6
0.5
0.4
0.3
0.2
0.1
0

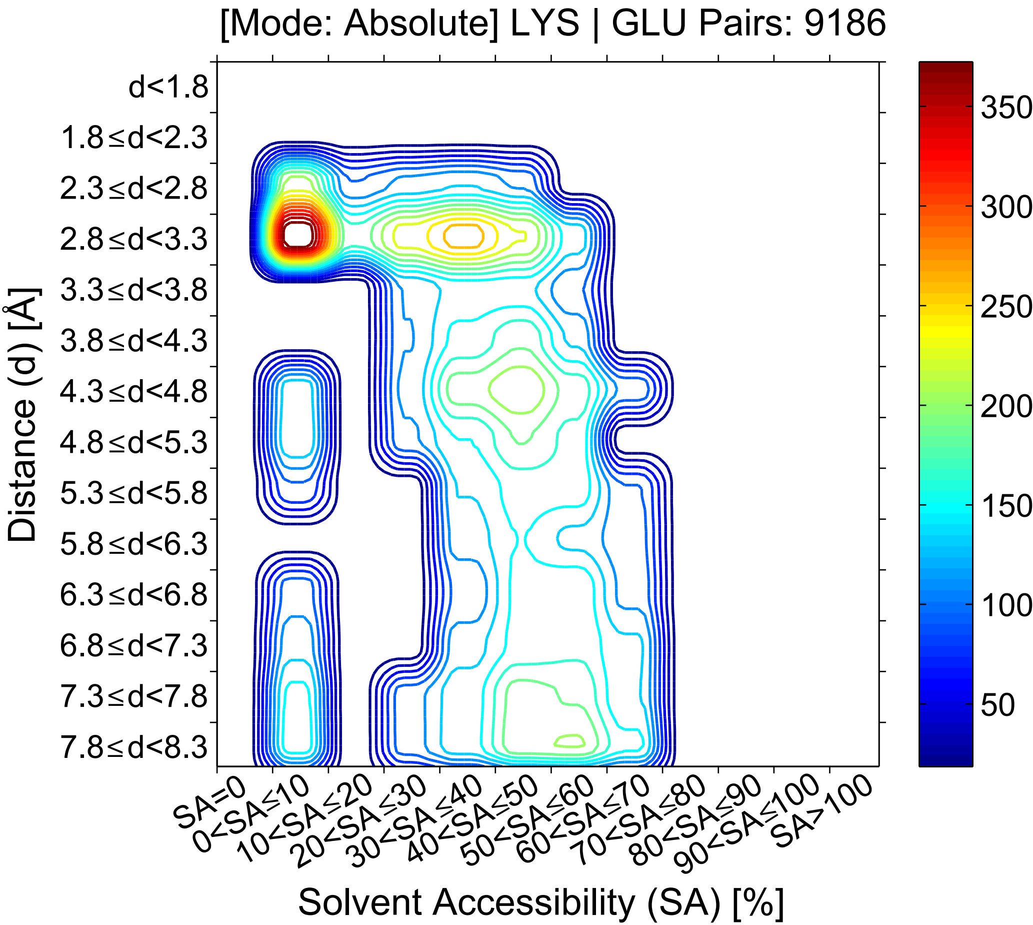
[Mode: Absolute] LYS | GLU Pairs: 9186
d<1.8
1.8≤d<2.3
2.3≤d<2.8
2.8≤d<3.3
3.3≤d<3.8
3.8≤d<4.3
4.3≤d<4.8
4.8≤d<5.3
5.3≤d<5.8
5.8≤d<6.3
6.3≤d<6.8
6.8≤d<7.3
7.3≤d<7.8
7.8≤d<8.3
Distance (d) [Å]
SA=0
0<SA≤10
10<SA≤20
20<SA≤30
30<SA≤40
40<SA≤50
50<SA≤60
60<SA≤70
70<SA≤80
80<SA≤90
90<SA≤100
SA>100
Solvent Accessibility (SA) [%]
350
300
250
200
150
100
50

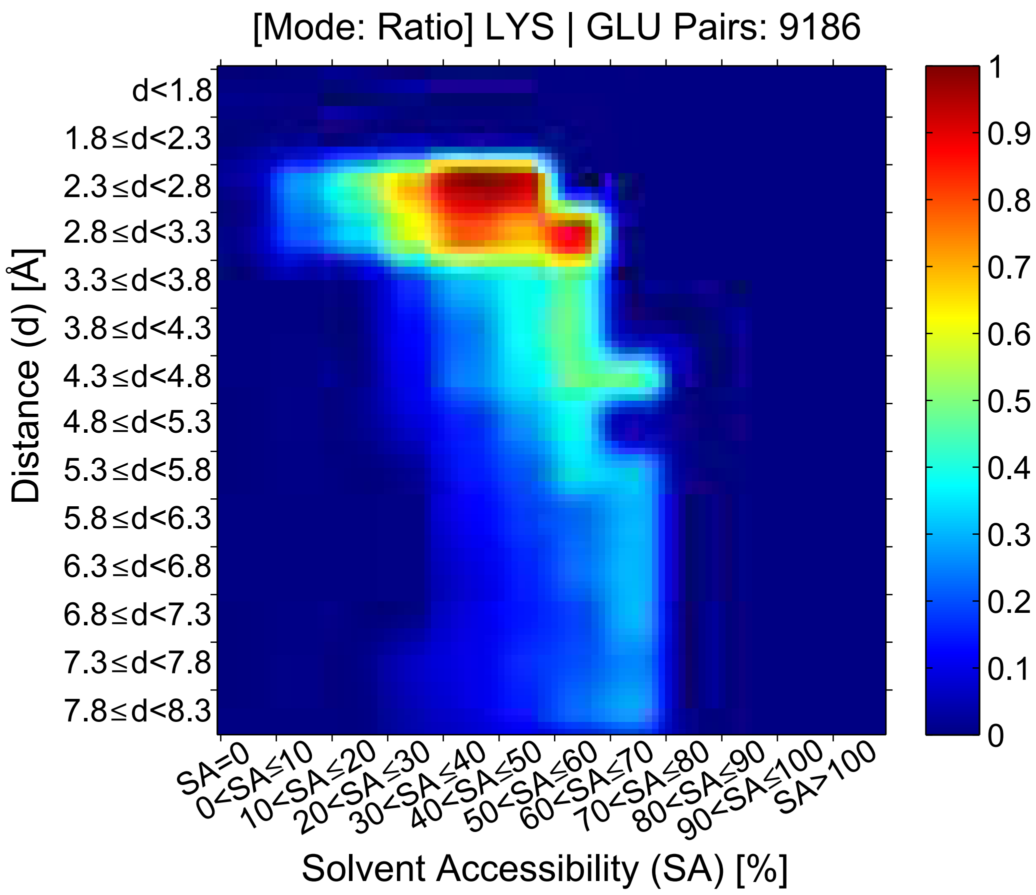
[Mode: Ratio] LYS | GLU Pairs: 9186
Distance (d) [Å]
d<1.8
1.8≤d<2.3
2.3≤d<2.8
2.8≤d<3.3
3.3≤d<3.8
3.8≤d<4.3
4.3≤d<4.8
4.8≤d<5.3
5.3≤d<5.8
5.8≤d<6.3
6.3≤d<6.8
6.8≤d<7.3
7.3≤d<7.8
7.8≤d<8.3
SA=0
0<SA≤10
10<SA≤20
20<SA≤30
30<SA≤40
40<SA≤50
50<SA≤60
60<SA≤70
70<SA≤80
80<SA≤90
90<SA≤100
SA>100
Solvent Accessibility (SA) [%]
1
0.9
0.8
0.7
0.6
0.5
0.4
0.3
0.2
0.1
0
